# Introduction to Radiobiology of Targeted Radionuclide Therapy

**DOI:** 10.3389/fmed.2015.00012

**Published:** 2015-03-17

**Authors:** Jean-Pierre Pouget, Catherine Lozza, Emmanuel Deshayes, Vincent Boudousq, Isabelle Navarro-Teulon

**Affiliations:** ^1^Institut de Recherche en Cancérologie de Montpellier, Montpellier, France; ^2^INSERM, U1194, Montpellier, France; ^3^Université de Montpellier, Montpellier, France; ^4^Institut régional du Cancer de Montpellier, Montpellier, France

**Keywords:** radiobiology, bystander effects, radioimmunotherapy, targeted radionuclide therapy

## Abstract

During the last decades, new radionuclide-based targeted therapies have emerged as efficient tools for cancer treatment. Targeted radionuclide therapies (TRTs) are based on a multidisciplinary approach that involves the cooperation of specialists in several research fields. Among them, radiobiologists investigate the biological effects of ionizing radiation, specifically the molecular and cellular mechanisms involved in the radiation response. Most of the knowledge about radiation effects concerns external beam radiation therapy (EBRT) and radiobiology has then strongly contributed to the development of this therapeutic approach. Similarly, radiobiology and dosimetry are also assumed to be ways for improving TRT, in particular in the therapy of solid tumors, which are radioresistant. However, extrapolation of EBRT radiobiology to TRT is not straightforward. Indeed, the specific physical characteristics of TRT (heterogeneous and mixed irradiation, protracted exposure, and low absorbed dose rate) differ from those of conventional EBRT (homogeneous irradiation, short exposure, and high absorbed dose rate), and consequently the response of irradiated tissues might be different. Therefore, specific TRT radiobiology needs to be explored. Determining dose–effect correlation is also a prerequisite for rigorous preclinical radiobiology studies because dosimetry provides the necessary referential to all TRT situations. It is required too for developing patient-tailored TRT in the clinic in order to estimate the best dose for tumor control, while protecting the healthy tissues, thereby improving therapeutic efficacy. Finally, it will allow to determine the relative contribution of targeted effects (assumed to be dose-related) and non-targeted effects (assumed to be non-dose-related) of ionizing radiation. However, conversely to EBRT where it is routinely used, dosimetry is still challenging in TRT. Therefore, it constitutes with radiobiology, one of the main challenges of TRT in the future.

## Introduction

This article, which is part of the inaugural series for the launch of Frontiers in Nuclear Medicine, will discuss some of the main challenges of radiobiology in targeted radionuclide therapy (TRT). Investigating radiobiology and performing accurate dosimetry will contribute to the improvement of the therapeutic efficacy of TRT, especially in the case of solid tumors.

Radiobiology explores the biological effects of radiations. This research field was created following the description of the first cases of cutaneous erythema associated with the clinical use of radiation at the beginning of the twentieth century. The use of X-rays to treat patients with cancer was first experimented by V. Despeignes in Lyon in 1896, 6 months after their discovery by W. Roentgen (1). As early as 1902, the ability to quantify the delivered radiation dose (dosimetry) and to establish the dose–effect relationship led to a significant improvement of the patients’ outcome. In 1919, dose fractionation started to be investigated by C. Regaud, at the Curie Institute (Paris), who described how to treat tumors with high absorbed doses, while protecting healthy tissues (2).

Most of what we know about radiobiology concerns external beam radiation therapy (EBRT). Particularly, the therapeutic efficacy of low (X and γ rays, electrons) and high linear energy transfer (LET) radiation (neutrons, Auger electrons, protons, alpha-particles, and heavy ions) has been extensively investigated. This has been accompanied by the development of new techniques and technologies for dose delivery to the tumor (3). Besides total dose and LET, the biological effects of radiation depend on the absorbed dose rate, absorbed dose fractionation, tissue oxygenation, and volume of irradiated tissue. In addition, the cell response to radiation is highly dependent on the nature of the irradiated tissue (genetic background, cell proliferation rate) and its microenvironment.

## Cellular Radiobiology

### Initial events

**Figure 1:**
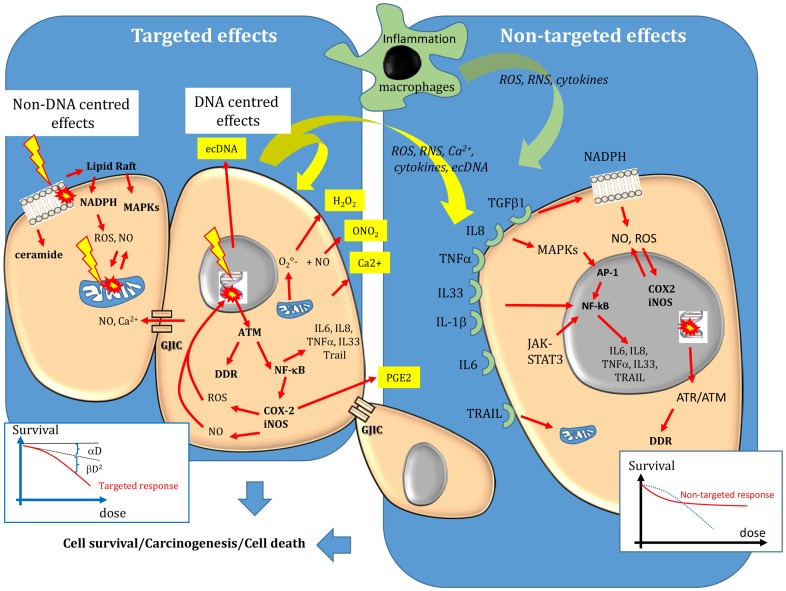
**Targeted and non-targeted biological effects in conventional external beam radiotherapy**. Targeted effects are caused by one or more particles traversing irradiated cells and can be divided in DNA and non-DNA-centered effects. Non-targeted effects describes the effects observed in cells that have not been directly traversed by particles but that are close to irradiated cells, as well as long-distance effects. DNA, mitochondria, and the cell membrane are the main sensitive targets of radiation. Following targeted and non-targeted effects, cells can survive (lesions are effectively repaired), they can die (lesions are not repaired) or they can be transformed. The dose–effect relationship of targeted effects is commonly fitted by linear or linear-quadratic models. A saturation of the response to non-targeted effects has been described. For more details, see the main text.

Ionizing radiations interact with biological substrates through direct and indirect mechanisms (4). Direct effects involve one-electron oxidation reactions, while indirect effects are mediated through water dissociation, leading to the production of reactive oxygen species (ROS), such as superoxide radicals $\left(O_{2}^{\cdot -}\right)$ and hydrogen peroxide (H_2_O_2_), the precursors of the highly damaging hydroxyl radicals (^⋅^OH). Noteworthy, these ROS are similar to those produced by endogenous sources, such as the mitochondrial oxidative metabolism (leading to $O_{2}^{\cdot -}$ formation during oxygen reduction), the plasma membrane-bound nicotinamide adenine dinucleotide phosphate [NAD(P)H] oxidases and lipoxygenases (5–7) and peroxisomes (formation of H_2_O_2_). Massive production of ROS and of reactive nitrogen species (RNS) is also mediated by activation of cyclooxygenase-2 (COX-2) and inducible nitric oxide synthase (iNOS), for instance, following induction of transcription factors involved in the inflammatory response, such as nuclear factor kappa B (NF-κB) or activator protein-1 (AP-1) (8). NF-κB is activated by ataxia telangiectasia mutated (ATM), the main protein involved in DNA damage recognition. COX-2 leads to production of prostaglandin-E2 and ROS that are released in the intra- and extra-cellular medium (9) and contribute to the inflammatory responses (8) (Figure 1). Activation of iNOS leads to the formation of nitric oxide (NO) that can react with superoxide anion to form RNS, such as peroxynitrite $\left({\mathrm{ONO}}_{2}^{-}\right)$ (10–12). ${\mathrm{ONO}}_{2}^{-}$ and NO are produced by macrophages during inflammatory reactions, but they are also released by irradiated cells (12). ${\mathrm{ONO}}_{2}^{-}$ can generate many of the degradation products observed with ^⋅^OH (9). Moreover, differently from ^⋅^OH that is very reactive and diffuses for only about 4 nm, ${\mathrm{ONO}}_{2}^{-}$ can diffuse easily within cells and its highly oxidizing protonated form (ONOH) can cause DNA damage, cell death as well as protein and lipid peroxidation. H_2_O_2_ and NO can diffuse between cells (4).

Therefore, ROS and RNS participate in physiological processes including cell signaling, immune response, inflammation, apoptosis, and cell growth, and also in the cell response to radiation (8). These endogenous and exogenous reactive species can cause cellular damage, when imbalance occurs between their production and their destruction by the cell enzymatic and non-enzymatic defense systems. For instance, $O_{2}^{\cdot -}$ can be reduced to H_2_O_2_ by the enzyme superoxide dismutase. H_2_O_2_ can in turn be reduced to water by the catalase or glutathione peroxidase enzymes, or can be used, in the presence of metal ions, such as Fe^2+^, to produce ^⋅^OH through the Fenton reaction. Superoxide dismutase, catalase, and glutathione peroxidase are part of the enzymatic defense system developed by cells to keep the level of these endogenous ROS as low as possible. Several intracellular components, particularly glutathione, urates, bilirubin, and vitamin E and C, can also act as radical scavengers. When the balance is tilted in favor of reactive species, all cell compartments (cell membrane, mitochondria, and particularly the nucleus) and constituents (DNA, lipids, and proteins) may be harmed and their functions altered.

### DNA damage

Radiation-induced oxidative DNA damage resulting from ^⋅^OH attack (indirect effect, water radiolysis) or from one-electron oxidation (direct effect) includes single-strand (SSB) and double-strand DNA breaks (DSB), DNA base damage (oxidized and abasic sites), and DNA–DNA or DNA–protein crosslinks.

The main reactions of radiation-induced radicals with DNA are hydrogen abstraction from deoxyribose molecules by ^⋅^OH (mainly from the C’ carbon of the sugar moiety) and ^⋅^OH addition to the π bonds of the bases [for reviews see Ref. (4, 13, 14)]. It is estimated that about 80% of ^⋅^OH radicals react with bases and the remaining 20% with sugar moieties. Hydrogen abstraction from the C2 of the sugar moiety can result in base loss or SSB formation through phosphate elimination. SSBs can also be produced after ^⋅^OH reaction with pyrimidine bases. DSBs can be caused by the attack of one or two ^⋅^OH radical species. However, it is very unlikely that a segment of a few DNA bases will be affected by two independent radiation events (between 10^–6^ and 10^–4^ Gy^–1^ for direct and indirect effects, respectively). Indeed, at biologically relevant doses, it is more probable that a single radiation track might induce ionization on opposite strands in close proximity. Therefore, DSBs can be produced after DNA denaturation consecutive to two SSBs occurring within a 20-bp stretch.

DNA damage incidence is proportional to the absorbed dose and is quantified per Gy and per cell, after exposure to low and high LET radiation (15). About 40 DSBs are formed per cell per Gy of low-LET radiation. DNA base lesions are the most abundant (about 1000/cell/Gy) and fourteen types of oxidative purine and pyrimidine DNA damage have been detected in cellular DNA, among which 8-oxo-7,8-dihydroguanine (8-oxoGua) and 5,6-dihydroxy-5,6-dihydrothymine (thymine glycol) are the most frequent (4). Although DSB frequency is quite low, cell survival and mutagenesis are highly dependent on DSB spatial distribution. About 150 DNA–protein and 30 DNA–DNA crosslinks are also estimated to be produced per Gy and per cell (15).

Accumulation of DNA damage can lead to even more complex lesions called locally multiply damaged sites (LMDS), also known as oxidative clustered DNA lesions (OCDLs) (16–18). LMDS occur when more than two lesions are produced within one or two helices, i.e., within 20 bp. These clustered DNA lesions include complex SSBs and also simple or complex DSBs. They may include up to 10 lesions in the case of low-LET radiation and even more complex damage with high LET radiation. Thus, although low and high LET radiation produce the same DNA lesions, their yield and spatial distribution are different.

### DNA damage repair systems

Cells have developed DNA damage repair (DDR) systems against DNA lesions. DNA damage activates sensor systems, such as ATM, Ataxia Telangiectasia, and Rad 3-related (ATR), which in turn induce signaling pathways involved in the cell response to radiation, including cell cycle arrest, DNA repair, or cell death (19–22). DNA base damage is mostly repaired by the base excision repair (BER) mechanism and to a lesser extent by the nucleotide excision repair (NER) mechanism (23, 24). DSB repair schematically involves two major recombination pathways. The first one is the homologous recombination (HR) system that is active specifically in the S and G2 phases of the cell cycle (25). It is an error-free mechanism because undamaged DNA (the sister chromatid) is used as template for DNA repair. The second mechanism is the non-homologous end-joining (NHEJ) system, which is active mainly in the G1 phase of the cell cycle (26). Although NHEJ is the main repair pathway used by irradiated cells, it is thought to be error-prone because the broken DNA ends are ligated without the need of an undamaged, homologous template.

When accurately repaired, DNA lesions have no effect on cell survival or in daughter cells. However, if the lesions are too complex or abundant, or if the cell DNA repair machinery is deficient, not all DNA lesions will be completely or correctly repaired. Unrepaired/misrepaired DNA damage can cause the formation of chromosomal aberrations when cells progress through the cell cycle, leading to mitotic catastrophe (cell death during mitosis) or to programed cell death (known as apoptosis). Not all unrepaired lesions are lethal for the cell. In this case, they may be passed to the daughter cells and could lead to mutations, genomic instability and eventually to cancer development (Figure 1).

### A DNA-centered approach

According to the DNA-centered view of radiation-induced damage, unrepaired DNA lesions are the lethal event leading to cell death. The concerned DNA lesions are mainly DSBs, but more complex damage, involving OCDLs, may also constitute lethal lesions. As the aim of radiation therapy is to kill tumor cells or at least to prevent their division, the clonogenic assay, which was developed by Puck and Markus in 1956 to investigate the ability of a cell to form a new colony, has become the reference technique for assessing the cell response to radiation (27). This assay shows that the clonogenic survival of irradiated cells decreases exponentially as a function of the mean absorbed dose. Plotted on a graph, the survival logarithm can be experimentally fitted by a linear or a linear-quadratic regression model, depending on whether high or low-LET radiation is used. The linear part (αD) of the equation corresponds to single-hit killing events, while the quadratic part (βD^2^) requires two hits to kill cells (Figure 1). Thus, the overall cytotoxic effect is due to the sum of single and double hit events. This interpretation of cell cytotoxicity corresponds to the so-called target theory, which is an essential concept for understanding radiation biology. The fundamental principle of the target theory is that inactivation of the target(s) by a lethal event requires the cells to be crossed by radiation. The shape of the survival curve can be affected by the tissue radiation sensitivity that is defined by the α/β ratio and represents the cell capacity to repair damage. Radiation sensitivity, which was first hypothesized by Bergonié and Tribondeau in 1905 (28), is essentially explained by the patient’s genetic background concerning the DNA repair enzymes, antioxidant defenses and tissue proliferation. For instance, patients with pathologies like Ataxia Telangiectasia, Xeroderma Pigmentosum, Cockayne, or Nijmegen breakage syndromes have defects in proteins involved in DNA DSBs repair and show hypersensitivity to radiation (29–31). The shape of the survival curves is also affected by the LET, tissue hypoxia and dose fractionation, as shown in the 60s by Barenden and colleagues (32–36). The relevance of the α/β ratios determined *in vitro* in human cell lines for understanding the *in vivo* values has been extensively reviewed (37, 38).

## New Paradigms in Radiation Biology: Non-DNA Centered and Non-Targeted Effects

For about a century, the paradigm of radiation biology has been that the biological effects of ionizing radiation occur only in the nucleus of cells crossed by particles and that cell death is strictly due to unrepaired or misrepaired DNA. Therefore, the biological effects of ionizing radiation should be strictly related to the energy absorbed by the tissues and the survival of irradiated cells, expressed as a function of the dose, should be strictly fitted by a linear or linear-quadratic curve explained by DNA hits. However, such DNA-centered approach is not fully satisfying. For instance, it cannot explain hypersensitivity to low doses and radiation sensitivity syndromes associated with mutation of cytoplasmic proteins. In addition, studies in cells, animal models, or patients treated by radiotherapy reported that biological effects could be observed also when only the cell cytoplasm was irradiated (known as non-DNA-centered effects) and in non-irradiated areas (known as non-targeted effects or bystander effects) (39–42). As early as 1922, the release of stress mediators in the serum of irradiated mice (43) or in blood samples from irradiated patients (44–46) was associated with long-distance bystander effects called abscopal effects. The concept of bystander effects emerged again in 1992 when Nagasawa and Little reported that in monolayer cell cultures exposed to alpha micro-beams, sister chromatid exchanges were observed in 30% of cells, although <1% of cells were crossed by particles (47). Since then, many studies have investigated the origin and nature of radiation-induced bystander effects that are defined as biological effects occurring in the neighborhood of irradiated cells (47).

Bystander effects include mutations, clastogenic effects, cell death, apoptosis, and cell transformation (6). They mainly occur after low dose (<1 Gy) or low dose-rate irradiation, although they have been observed in EBRT also after high absorbed dose (10 Gy). They involve signaling from irradiated cells toward non-irradiated cells. Specifically, stress mediators are transmitted to bystander cells by cell–cell interactions through gap junction intercellular communication (GJIC) when cells are in contact and the molecules are small (<1500 Da) (Figure 1), or by the release of soluble damage/stress signals that may have distant biological effects (abscopal effect) (6, 48–50). These mediators can be ROS/NO, cytokines (interleukin 8, interleukin 6, tumor necrosis factor, and interleukin-33), Ca^2+^, or extracellular DNA (ecDNA). They are produced by irradiated cells and are released in the extracellular environment (39), thus inducing oxidative stress in neighboring cells/tissues. However, they can be active also within the cell in an autocrine way. They can also activate immune cells (for instance, macrophages and T lymphocytes) that, in turn, release cytokines, leading to iNOS induction, and NO formation (51). Therefore, the inflammatory and radiation responses share common mechanisms to promote and perpetuate a harmful inflammatory/oxidative stress environment. This new paradigm also highlights the role of the tumor (or healthy tissue) microenvironment in the radiation response. The finding that ROS scavengers, such as DMSO, abolish the bystander response indicates that oxidative stress plays a major role in this phenomenon (52). While ^⋅^OH have a short life and interact within few nm, other species, such as H_2_O_2_ and NO, can migrate across the plasma membrane and cause oxidative damage in neighboring cells. Moreover, other systems leading to sustained intercellular production of reactive radicals can be activated in neighboring cells after cytokine release by irradiated cells or binding of immune cells to bystander cells. Interleukin-1β (IL-1β), tumor necrosis factor-α (TNF-α), and interleukin-33 (IL-33), for instance, activate NF-κB that is involved in the expression of the COX-2 and iNOS genes, which participate in the inflammatory response and the local production of ROS and NO, respectively (Figure 1). Furthermore, TNF-α, interleukin 8, and transforming growth factor 1 b (TGFβ-1b) can activate the mitogen-activated protein kinase (MAPK) pathways [extracellular signal-related kinase (ERK), c-JUN N-terminal kinase (JNK), and p38] that participate in COX-2 and iNOS up-regulation (11, 53). Interleukin 6 is released by irradiated macrophages and activates Janus-kinase 2 (JAK2)-signal transducer and activator of transcription 3 (STAT3). STAT3 contributes to NFκ-B retention in the nucleus and thereby in the induction of COX-2 and iNOS expression (54, 55). TGFβ-1 secreted by irradiated cells can also activate NADPH oxidase, which is located at the cell membrane and is involved in bystander ROS and NO production. Therefore, bystander factors are involved in the long-term production of reactive radicals in a feed-forward and self-sustaining fashion and in the creation of an inflammatory environment, leading to the recruitment of immune cells (52). Noteworthy, the increase in intracellular oxidative stress leads to mitochondrial dysfunction that can further exacerbate oxidative processes by releasing ROS and RNS.

Finally, it has been shown that different signaling pathways are involved in DNA repair, depending on whether DNA damage is produced directly by irradiation, or indirectly by oxidative stress-induced bystander effects (56). Moreover, while targeted biological effects increase with the dose in EBRT, a saturation response is observed when non-targeted effects are involved, and above a certain dose, no additional effect is observed (Figure 1).

## New Paradigms in Radiation Biology: Extra-Nuclear Targets

### Cell membrane

Although most attention has been focused on DNA as the main radiation target, the idea that the membrane could also be an important target was suggested in 1963 by Alper et al. (57). The cell membrane is now recognized as a key player in the radiation-induced biological effects. Its role in the cellular response to radiation may be explained by its function in many signaling pathways, including apoptosis (58–60). Radiation-induced hydroxyl radical molecules can attack not only nuclear DNA but also polyunsaturated fatty acid residues of membrane phospholipids. This results in the formation of malonedialdehyde or 4-hydroxynonenal that can induce DNA–protein crosslinks (58). Radiation can also cause activation of acid sphingomyelinase that hydrolyzes sphingomyelin in the cell membrane to produce ceramide and phosphorylcholine (59–62). Ceramide is a second messenger of apoptosis and is also involved, when associated with cholesterol, in the formation of ceramide-enriched platforms (also known as lipid rafts) containing signaling and transport proteins. These platforms play a central role in cellular functions such as cell signaling and trafficking. Specifically, plasma membrane activation stimulates diverse signaling pathways that are mediated by the MAPK superfamily, including ERK1/2, JNK, and p38. Moreover, lipid rafts contain NADPH oxidase that is involved in the sustained ROS/RNS production by bystander cells. NADPH can be activated by TGFβ secreted by irradiated cells after activation of cell membrane receptors by cytokines (TGFβ, TNFα, interleukins) and Ca^2+^ ion channels. These indirect effects can be partially inhibited by antioxidants, such as vitamins E and C, and by the enzymes superoxide dismutase, catalase, and glutathione peroxidase.

### Mitochondria

Mitochondria are central cell organelles. They are involved in cell respiration by reducing O_2_ into $O_{2}^{\cdot -}$ during ATP production and constitute one of the main source of endogenous ROS and RNS (8, 63). They also play a role in radiation-induced cell signaling pathways, such as apoptosis (64). Indeed, one of the critical events is the change in mitochondrial membrane potential leading to leakage and release in the cytosol of pro-apoptotic proteins, including cytochrome C and apoptosis-inducing factor (10). This may be due to high ROS and NO production in response to direct irradiation or non-targeted effects. Moreover, ROS, such as superoxide anions, released by mitochondria can contribute to intracellular oxidative stress and non-targeted effects through their conversion into diffusible H_2_O_2_ molecules (52).

Mitochondrial DNA can also be altered after ROS attack or by direct radiation effects (65–67). Mitochondrial DNA is very sensitive to oxidative stress because it is not protected by histones, and mutations or deletions have been observed. When they concern the genes coding for mitochondrial ATPase, NADH dehydrogenase complex I and cytochrome c oxidase, they can lead to defects in the mitochondrial metabolism and the DNA repair efficiency as well as to the increase in ROS level. These effects have been observed both in directly irradiated cells and in non-targeted cells.

## Targeted Radionuclide Therapy

**Figure 2:**
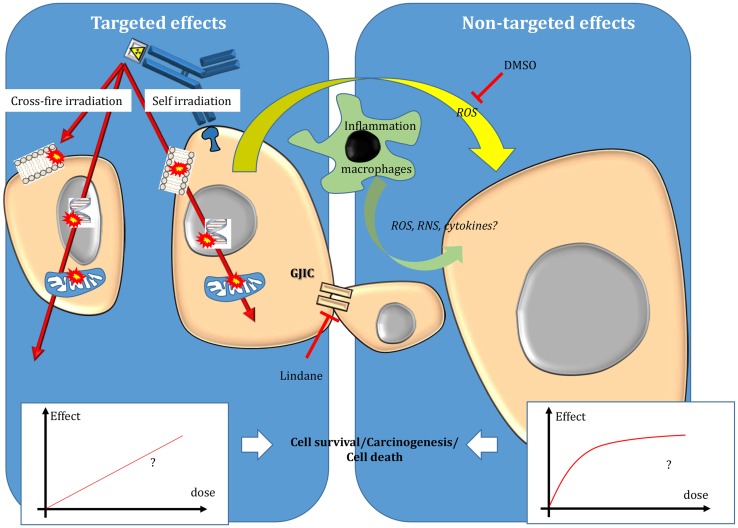
**Targeted and non-targeted effects in targeted radionuclide therapy**. Targeted effects are caused by one or more particles crossing irradiated cells and can be due to self-irradiation and cross-fire irradiation. Non-targeted effects include effects observed in cells close to irradiated cells and also long-distance effects. The nature of the dose–effect relationship resulting from targeted and non-targeted effects needs to be determined. For more details, see the main text.

Beside EBRT improvements, TRT has emerged as an attractive approach for treating tumors during the twentieth century. In TRT, a radionuclide is coupled to a vector [for instance, monoclonal antibodies (mAbs) or peptides] directed against cancer cells or their environment to specifically irradiate only the tumor targets. Therefore, TRT is particularly attractive when conventional EBRT (CEBRT) cannot be used due to unacceptable toxicities toward healthy tissues. This is the case of disseminated disease, metastases, or tumors located in close vicinity of sensitive organs. Compared to chemotherapy, it offers the possibility to specifically target tumor cells, thereby reducing the side effects, and also to treat distant tumor cells (which cannot be directly reached by the drug) through cross-fire irradiation (i.e., energy deposition in cells that are not specifically targeted) (Figure 2).

Radionuclide therapy started in the 20–40s when injections of very simple chemical forms of a radionuclide (^131^I or ^32^P) were used to treat patients with differentiated thyroid or ovarian cancers, respectively. Other compounds have been largely employed in nuclear medicine, such as ^89^SrCl_2_, ^153^Sm-EDTMP, or ^186^/^188^Re–HEDP, for palliative treatment of bone metastases. Radio-embolization with ^90^Y microspheres has been used for the treatment of hepatocellular carcinoma. In 2013, ^223^Ra chloride (Xofigo^®^) was the first Food and Drug Administration (FDA)-approved unsealed α-emitting radiopharmaceutical for the treatment of patients with castration-resistant prostate cancer and metastatic bone disease. Radionuclides can also be bound to a vector that specifically targets tumor cells for TRT. Specific molecules, such as metaiodobenzylguanidine (mIBG) labeled with ^131^I for TRT of neuroblastoma and medullary thyroid cancer, have been progressively developed. Peptide receptor radionuclide therapy (PRRT) started to be assessed in the 70–80s and led to the development of ^90^Y-DOTATATE and ^90^Y-DOTATOC that are currently used for the treatment of neuroendocrine tumors. In radioimmunotherapy (RIT), antibodies against cancer cell antigens are used to target the radionuclide to cancer cells. In RIT, the radiation-induced biological effects may be combined with the antibody cytotoxic effect. The first RIT assays in patients using ^131^I-labeled polyclonal antibodies against cancer cell antigens were performed in 1953. The first clinical trials with mAbs (^131^I-labeled or ^67^Cu-labeled anti-HLA-DR mAbs) began in 1988 and ^131^I-labeled anti-CD20 antibodies were first used in patients with non-Hodgkin lymphoma (NHL) in 1993. Two radiopharmaceuticals for RIT have been approved by FDA (^90^Y-ibritumomab tiuxetan, Zevalin^®^, in 2002 and ^131^I–tositumomab, Bexxar^®^, in 2003) for the treatment of relapsed or refractory low-grade, follicular, or transformed B-cell lymphoma (68). NHL is undoubtedly the disease in which RIT has the highest success rates. Indeed, the overall response rate is between 60 and 83% in previously treated patients and of 95% in patients treated for the first time (69) compared to 56% in patients treated with rituximab immunotherapy (68). As the Zevalin^®^ and Bexxar^®^ activities administered to patients are currently based only on the patient’s weight (MBq/kg for Zevalin^®^) or on delivering 75 cGy to the whole body (Bexxar^®^), further improvements could be expected by planning patient-tailored treatments using a dosimetry approach.

On the other hand, TRT of solid tumors is more challenging, mainly because these tumors are more radiation-resistant than lymphomas. In such context, the approach consisting in one injection of the maximal tolerated activities of a radiopharmaceutical, based on the patient’s weight, is not sufficient and a more complex strategy is required (70–73) that takes into account both radiobiology and dosimetry data.

### TRT radiobiology specificity

Although particles emitted by radionuclides produce similar physical events (ionization/excitation) as those described in EBRT, TRT radiobiology cannot be strictly extrapolated from the radiobiology developed for EBRT.

**Figure 3:**
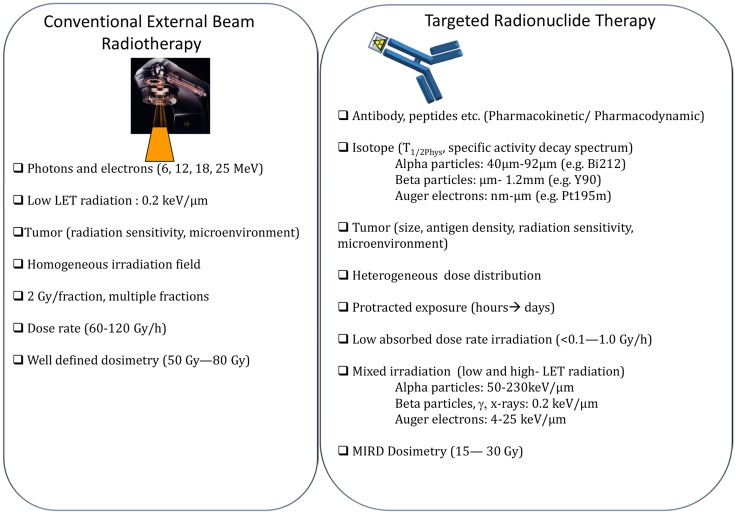
**Comparison of conventional external beam radiotherapy and targeted radionuclide therapy**.

The main differences between EBRT and TRT concern the absorbed dose-rate and the spatial energy deposit (Figure 3). CEBRT produces homogeneous irradiation at high absorbed dose rate (about 1–2 Gy⋅min^−1^) of low-LET X-rays that target all the cells in the field with a total absorbed dose of 40–80 Gy, administered in fractions of 2 Gy, five fractions per week. Conversely, TRT is characterized by low absorbed dose rate (<1 Gy⋅h^−1^) with protracted, heterogeneous, and mixed irradiation. Indeed, vectors can be coupled to radionuclides that emit beta, alpha, or Auger electrons, associated or not with X or γ rays. In addition, when using alpha particle emitters, the decay spectrum of daughter radionuclides should also be taken into account. Therefore, LET ranges from 0.2 keV/μm for beta, X, and γ rays and 4–25 keV/μm for Auger electrons to 50–230 keV/μm for alpha particles (Figure 3). The particle path length will be also variable: few nm–μm for Auger electrons, fifty to about one hundred μm for alpha particles and from μm to mm for beta particles. Finally, the distribution of radiolabeled molecules is generally non-uniform and leads to strong heterogeneity in activity distribution, whatever scale considered (subcellular, organ, or organism). Therefore, some territories will be irradiated while others may partly escape. This phenomenon is even more marked for short range emitters, such as alpha particle and Auger electron emitters, because the path length of the emitted particles produces low cross-fire irradiation (i.e., cells are crossed by particles emitted by radiolabeled vectors bound to neighboring cells) (Figure 2). However, the highly localized energy deposition associated with Auger electrons and alpha particles is also an attractive tool to investigate the biological effects of ionizing radiations at the subcellular scale and has shown the weaknesses of a completely DNA-centered conception of TRT effects. Indeed, we and others have demonstrated that extra-nuclear targets are also involved in targeted and non-targeted responses (74–78). The existence of non-targeted effects may affect the absorbed dose–effect relationship.

### Improving dosimetry in TRT

One difficulty when investigating TRT radiobiology is that in many preclinical and clinical studies, dosimetry is not available or is not fully accurate. Biological effects are often related to the activities injected in patients/animal models (Bq/kg) or added to the medium of cultured cells in *in vitro* experiments (Bq/mL). However, like for EBRT, dose assessment is, at least theoretically, the only reference parameter common to all forms of treatment involving radiation and the dose–effect relationship provides useful information on how radiations act. In TRT, the biological effects observed in patients/animal models or in cultured cells will depend on the used activity, its distribution within the different compartments (organs, cell compartments), its pharmacokinetics/dynamics, the type of emission, and the target geometry (organ, cells, nucleus) and also, like for EBRT, on the nature of the targeted tissue and its microenvironment.

Therefore, dosimetry is a requirement for the radiobiologist; however, determining the absorbed dose is not straightforward in TRT and is often considered a tedious and unattractive task. The approach commonly used is based on the medical internal radiation dose (MIRD) formalism (79):

DrT,TD=∑rSÃrs,TD SrT←rS

The MIRD formalism requires to determine the cumulative number of decays *Ãr_S_*, which is the time integrated activity (or total number of decays) occurring in a source region *r_S_* over the dose integration period *T_D_*, and the corresponding *S* values, which represent the absorbed dose in the target region *r_T_* per nuclear transformation in *r_S_*_._

#### Determining the activity distribution and *S* values

The reliability of the radiation dose estimates in preclinical and clinical TRT studies is directly related to the accuracy of the activity assessment at each time point over the considered period. For instance, in *in vitro* experiments, the uptake of radioactivity per cell is assessed at various intervals after exposure to the radiolabeled vector. For this, the radioactivity distribution within the cell population needs to be considered (80). Moreover, the subcellular distribution may be different depending on the targeting vector and on the final source (cell membrane, cytoplasm, organelles, or nucleus) according to the MIRD formalism. For animal experiments, several methodologies are now available. The standard method relies on the determination of the average radioactivity in different organs after animal sacrifice at various time points following administration of the radiopharmaceutical. This approach can be completed by digital autoradiography that provides information about the spatio-temporal distribution of radioactivity in tissue cryosections (81). The development of small animal imaging techniques (micro-SPECT-CT or micro-PET-CT) provides an attractive alternative method by reducing the number of needed animals and allowing longitudinal studies (82, 83). However, because of the inherent limits of these imaging techniques, the determination of radioactivity is limited to organs showing specific targeting. In the clinic, dosimetry is routinely used in EBRT; conversely, TRT dosimetry is still in its early days and it is much more difficult. Initially, conventional methods based on planar imaging were used to assess the radioactivity distribution. However, it is now clear that, due to the inherent heterogeneity of the distribution of a radiopharmaceutical in tissues, three-dimensional anatomical (CT or MRI) and functional [PET or single-photon emission CT (SPECT)] imaging approaches are required and that the *S* value must be calculated using Monte Carlo simulations. However, these techniques are much more demanding in terms of human and methodological resources and uncertainties in the radiation dose estimation may still arise from incomplete kinetic assays and low accuracy in volume measurements and *S* value calculation (84). In both clinical and preclinical studies, *S* values (i.e., the absorbed dose per decay) can be determined using Monte Carlo codes that allow following the radiation transport and scoring the energy deposit (85–87). For this purpose, the cell, organ, or organism geometry must be known (87, 88). This can be done by microscopic observation of cells *in vitro*, and by using phantoms or the information provided by CT scans for animal models and patients.

#### Determining dose–effect relationship

Unfortunately, very few preclinical studies are available to demonstrate the validity in TRT of the linear quadratic or linear dose–effect relationship, as commonly observed in EBRT. In clinical TRT studies, interesting and encouraging results on the correlation between tumor-absorbed dose and treatment efficacy have been highlighted (89, 90), although no strong dose–effect relationship has been shown even in lymphoma (91), which is the disease most frequently treated by TRT. Strigari et al. reported that among 79 radiotherapy studies investigating dosimetry, an absorbed dose–effect correlation was found in 48 (92). Yet, the existence of a dose–effect relationship (efficacy/toxicity) in TRT is still a matter of debate. Besides performing accurate dosimetry, the most relevant biological endpoints must also be identified. Indeed, the follow-up of tumor shrinkage in patients is difficult because TRT is mainly used to treat small-volume solid tumors or disseminated disease. Therefore, other parameters, such as the clinical response or progression-free survival, should be investigated as endpoints to evaluate TRT efficacy and the dose–effect relationship (90, 93).

The link between absorbed dose and associated toxicities in healthy tissues (based on the creatinine level for kidney toxicity, blood cell count for bone marrow toxicity, or liver functional parameters) has also been studied in several targeting models, including PRRT (94). Therapeutic regimens could then be based on the maximal absorbed dose tolerated by healthy tissues rather than on the dose delivered to the tumor.

### Investigating the “inverse dose rate-effect” phenomenon

In TRT, the absorbed dose rate depends on the physical half-life of the radionuclide, its specific activity and the vector pharmacokinetics (transit, uptake, and clearance). Therefore, irradiation is usually protracted from hours to days and the generally low absorbed dose-rate values (<1 Gy⋅h^−1^) are expected to give cells time to repair damage. As the clonogenic survival of cells decreases when the dose or dose rate increases, TRT efficacy should therefore be very low. However, this is not true and TRT therapeutic efficacy per Gy, when accurate dose determination is available, is higher than that of CEBRT (95, 96). The observation that low absorbed dose rates are ultimately more cytotoxic per Gy than doses delivered at high dose rate is defined as the inverse dose rate-effect. It has been described also after low dose of EBRT and could contribute to the hypersensitivity to low doses (95, 97–99). Several hypotheses have been proposed to explain this effect, including TRT-mediated synchronization of cells in a radiosensitive cell cycle phase or defects in the detection of low levels of DNA damage. However, these mechanisms cannot be generalized (78) and TRT biology still needs to be investigated for each TRT situation. Models and notions have been developed to reduce the discrepancies between the theoretical dose–effect relationship and the real biological effects measured in human patients using clearly identified endpoints (creatinine values, for example). The notion of biological effective dose (BED) has been introduced to take into account the biological effects of low absorbed dose rates and the repair capacities allowed by protracted irradiation, as it is done for fractionation in EBRT. The concept of equivalent uniform biologically effective dose (EUBED) is used to take into account the dose distribution heterogeneity (100). However, these improvements might not be enough if all the aspects of radiobiology of the tissues treated by TRT have to be taken into account. For example, in most TRT dosimetric approaches, α/β are still extrapolated from EBRT data and these values should be confirmed (101, 102).

### Investigating non-targeted effects in TRT

As non-targeted effects have been described mostly after low doses of EBRT, their contribution in TRT (50, 103–106) should be more prominent because the dose is generally delivered at low dose rates (104). Therefore, the final TRT cytotoxicity could be the sum of both targeted effects (described by the absorbed dose–effect relationship) and non-targeted effects, which are likely to be described by the lack of absorbed dose–effect relationship and a saturated response. Therefore, the nature of the global absorbed dose–effect relationship, resulting from both phenomena, needs to be investigated.

Although the analysis of non-targeted effects in TRT is more challenging than in EBRT, several *in vitro* and *in vivo* studies have shown that beta-emitting particles, such as those released by ^3^H incorporated into DNA (thymidine (3H-dThd) or by ^131^I incorporated in metaiodobenzylguanidine (^131^I-MIBG), and Auger electrons emitted by ^125^I coupled to deoxyuridine (^125^IUdR) (26) or to antibodies (106) could lead to non-targeted effects. This was also observed when alpha-particle emitters (^213^Bi, ^211^At) were used to radiolabel mAbs (74, 75) or MIBG (105, 107), respectively. Moreover, drugs that interfere with gap junctions (for instance, lindane) or that scavenge ^⋅^OH radicals (DMSO) can abrogate the non-targeted response in a cell model of TRT (108).

The relative contribution of non-targeted effects, compared to the direct effects of radiation, may depend on the TRT nature and specifically on the absorbed dose rate and LET (105–107). One could expect TRT to behave like EBRT for long-range particles, thereby producing homogenous irradiation and for situations with high tumor uptake of radioactivity, thereby producing high dose–rate irradiation. However, this needs to be further confirmed.

## Conclusion

Although the methodology for exploring radiation biology in conventional EBRT is well established, a specific method dedicated to TRT needs to be developed. This involves a solid dosimetry approach that takes into consideration the different TRT situations and that may become the reference. Moreover, the absorbed dose and absorbed dose rate are likely to be the critical parameters in the radiobiological response to TRT. The relative contribution of targeted and non-targeted effects in the organ and tissue responses to TRT needs also to be determined.

Preclinical experiments offer the possibility to study in simple models how TRT acts on cells and tissues with the aim of identifying the specific molecular and cellular mechanisms, as it has been done in EBRT. They might provide ways for improving TRT by taking into account both radiobiology and dosimetry to shift from a radioactive chemotherapy approach toward true targeted radiotherapy.

## Conflict of Interest Statement

The authors declare that the research was conducted in the absence of any commercial or financial relationships that could be construed as a potential conflict of interest.
